# Supplementary material for: Chikungunya in Indonesia: Epidemiology and diagnostic challenges
Source: PLoS Negl Trop Dis. 2020 Jun 1;14(6):e0008355. doi: 10.1371/journal.pntd.0008355 (PMC7289446; doi:10.1371/journal.pntd.0008355)
Supplement: S1 Table — (DOCX) [file pntd.0008355.s002.docx]

**Supporting Table 1.**  Clinical and hematology profiles, treatment and outcomes in pediatric and adult ACI cases

|  | **Total** | **Pediatric**  **(N=17)** | **Adult**  **(N=23)** |
| --- | --- | --- | --- |
| **Demographics** | | | |
| Age, median (range) years | 20.4 (1-83.1) | 5.7 (1-16.3) | 28.1 (20.1-83.1) |
| Female, N (%) | 16 (40) | 9 (52.9) | 7 (30.4) |
| **Fever** | | | |
| Days ill, median (IQR) | 2 (1-6) | 1 (2-5) | 2 (1-6) |
| Temperature (°C), median (IQR) | 38.9 (36-41.2) | 39 (37-39.9) | 38.9 (36-41.2) |
| **Symptoms, N (%)** | | | |
| General symptoms | | | |
| Anorexia | 10 (25) | 7 (41.2) | 3 (13) |
| Chills | 9 (22.5) | 4 (23.5) | 5 (21.7) |
| Lethargy | 10 (25) | 3 (17.6) | 7 (30.4) |
| Headache | 18 (45) | 4 (23.5) | 14 (60.9) |
| Neurological | | | |
| Convulsion | 5 (12.5) | 5 (29.4) | 0 |
| Decrease of consciousness | 1 (2.5) | 0 | 1 (4.3) |
| Respiratory | | | |
| Cough | 9 (22.5) | 5 (29.4) | 4 (17.4) |
| Haemoptysis | 1 (2.5) | 1 (5.9) | 0 |
| Runny nose | 2 (5) | 2 (11.8) | 0 |
| Epistaxis | 2 (5) | 2 (11.8) | 0 |
| Shortness of Breath | 3 (7.5) | 2 (11.8) | 1 (4.3) |
| Gastrointestinal | | | |
| Diarrhea | 5 (12.5) | 1 (5.9) | 4 (17.4) |
| Nausea | 26 (65) | 9 (52.9) | 17 (73.9) |
| Vomiting | 14 (35) | 8 (47.1) | 6 (26.1) |
| Epigastric Pain | 5 (12.5) | 1 (5.9) | 4 (17.4) |
| Musculoskeletal | | | |
| Arthralgia | 18 (45) | 4 (23.5) | 14 (60.9) |
| Myalgia | 12 (30) | 5 (29.4) | 7 (30.4) |
| Skin rash | 6 (15) | 2 (11.8) | 4 (17.4) |
| Petechiae/ ecchymosis | 4 (10) | 3 (17.6) | 1 (4.3) |
| **Laboratory findings , N (%)** | | | |
| Leukocytes* |  |  |  |
| Leukopenia | 4 (10) | 1 (5.9) | 3 (13) |
| Normal | 31 (77.5) | 14 (82.4) | 17 (73.9) |
| Leucocytosis | 5 (12.5) | 2 (11.8) | 3 (13) |
| Lymphopenia | 19/35 (54.3) | 9/16 (56.3) | 10/19 (52.6) |
| Normal lymphocytes | 13/35 (37.1) | 5/16 (31.3) | 8/19 (42.1) |
| Lymphocytosis | 3/35 (7.5) | 2/16 (12.5) | 1/19 (5.3) |
| Platelets |  |  |  |
| Normal thrombocytes* | 36 (90) | 17 (100) | 19 (82.6) |
| Thrombocytopenia (≤150,000/mm^3^) | 4 (10) | 0 | 4 (17.4) |
| **Treatment**, N | | | |
| Antimicrobial | 21 | 10  Amoxicillin (3)  Ampicillin (2)  Cefixime  Cefotaxime  Ceftriaxone  Chloramphenicol  Cotrimoxazole | 11  Ceftriaxone (7)  Amoxicillin  Cefixime  Ciprofloxacin  Levofloxacin |
| **Outcomes** | | | |
| Cured** | 31 (77.5) | 15 (88.2) | 16 (69.9) |
| Cured with sequelae*** | 9 (22.5) | 2 (11.8) | 7 (30.4) |
| Death before discharge | 0 | 0 | 0 |

*Adjusted by age ; **Cured : original illness is no longer present ; *** Sequelae : Lethargy, myalgia
