# Supplementary material for: Chikungunya in Indonesia: Epidemiology and diagnostic challenges
Source: PLoS Negl Trop Dis. 2020 Jun 1;14(6):e0008355. doi: 10.1371/journal.pntd.0008355 (PMC7289446; doi:10.1371/journal.pntd.0008355)
Supplement: Attachment — Submitted filename: RESPONSE to REVIEWERS - CHIKV.docx [file pntd.0008355.s004.docx]

**Response to reviewers’ comment**

Summary and General Comments

Reviewer #1: This paper reports on the testing of febrile cases in Indonesia for evidence of CHIKV infection (acute and historic). This paper represents a useful study that quantifies the frequency of CHIKV misdiagnosis as well as the history of CHIKV infection in Indonesia. My only major concern is the over-interpretation of secondary ACI.

**1) I was not convinced by the argument that two cases were probably (or ‘likely’) secondary ACI. This is important in the context of current vaccine development, which relies on immunological markers of lifelong protection. The authors rely on the presence of IgG as measured by an ELISA in an acute sample as evidence of probable secondary acute CHIKV infection. This is at best, weak evidence and should be downplayed. The presence of IgG in an acute sample could be due to cross-reactivity with other alphaviruses or faster than average development of IgG antibodies. I agree that it is an interesting observation but given the substantial uncertainty as to whether they are truly secondary infections, at a minimum I would refer to these as ‘potential/possible secondary ACI’. It should also be discussed in the context of cross-reactivity with other circulating alphaviruses (e.g., see Tesh et al., AJTMH 1975) and a study in the Philippines that showed that all cases of ACI in a Philippines cohort were among those with no detectable antibodies (as measured by more specific PRNTs rather than ELISAs) at baseline (Yoon et al., PLoS NTD).**

**Response:** We thank the reviewer for this important suggestion. We agree to change ‘probable’ or ‘likely’ to ‘possible’ throughout the text. We also agree to add in the discussion that cross-reactivity with other circulating alphaviruses is possible.

**Line 51 now read:** Two of the 40 cases were possibly secondary ACI.

**Line 168-173 now read:** Possible secondary ACI was inferred when the above criteria was met, and IgG was detected in acute specimens. Probable ACI was inferred when only CHIKV IgM was detected in the acute specimen, but increased index could not be confirmed due to lack of convalescent specimen. Probable ACI with possible secondary infection was inferred when RT-PCR was negative, and IgM sero-converted but OD in acute and convalescent specimens was equivalent.

**Line 240-241 now read:** In 2 cases, IgG was also detected in acute specimens, suggesting possible secondary ACI.

**Line 262- 264 now read:** In two probable ACI with possible secondary infection cases, IgM sero-converted or increased (0.8 to 3, and 2.4 to 3.3) with high index or slightly increased IgG (3.2 to 3.2 and 2.8 to 3.3).

**Line 329-330 now read:** Third, possible secondary ACI may occur.

**Line 409-423 now read:** The identification of two possible secondary ACI in Semarang and Surabaya support previous findings from Bandung [3]. There are several plausible explanations. First, recovery from infection may not engender lifelong immunity. Second, development of CHIK IgG in these patients may have been faster than average. Third, cross-reactivity with other alphaviruses (Ross River virus, Eastern equine encephalitis virus, Barmah-Forest virus, Mayaro virus) [38,39] is possible given that false positives have been reported with the IgG kit we employed when compared to an in-house ELISA assay used by the US-CDC in Panama [39]. However, there were no reports of the circulating Ross river viruses or other alphaviruses in Indonesia, except in Moluccas, which was not a site in our study [38,40]. Furthermore, a study in the Philippines, using the more specific PRNT assay, revealed that CHIK infection only occurred in 106 subjects who did not have CHIKV neutralizing antibodies in their acute samples [41]. Finally, it is also possible that the strains causing the first and second infections were different, which may occur with circulation of both Asian and ECSA genotypes in Indonesia [6].

References:

3.	Kosasih H, de Mast Q, Widjaja S, Sudjana P, Antonjaya U, Ma'roef C, et al. Evidence for endemic chikungunya virus infections in Bandung, Indonesia. PLoS Negl Trop Dis. 2013;7(10):e2483. doi: 10.1371/journal.pntd.0002483. PubMed PMID: 24205417; PubMed Central PMCID: PMCPMC3812099.

37.	Hassing RJ, Leparc-Goffart I, Tolou H, van Doornum G, van Genderen PJ. Cross-reactivity of antibodies to viruses belonging to the Semliki forest serocomplex. Euro Surveill. 2010;15(23). pii: 19588.

38.	Porter KR, Tan R, Istary Y, Suharyono W, Sutaryo, Widjaja S, Ma'Roef C, Listiyaningsih E, Kosasih H, Hueston L, McArdle J, Juffrie M. A serological study of Chikungunya virus transmission in Yogyakarta, Indonesia: evidence for the first outbreak since 1982. Southeast Asian J Trop Med Public Health. 2004;35(2):408-15.

39.	De Salazar PM, Valadere AM, Goodman CH, Johnson BW. Evaluation of three commercially-available chikungunya virus immunoglobulin G immunoassays. Rev Panam Salud Publica. 2017;41:e62.

40.	Tesh RB, Gajdusek DC, Garruto RM, Cross JH, Rosen L. The distribution and prevalence of group	A arbovirus neutralizing antibodies among human populations in Southeast Asia and the Pacific	islands. Am J Trop Med Hyg. 1975;24(4):664-75.

41.	Yoon IK, Alera MT, Lago CB, Tac-An IA, Villa D, Fernandez S, Thaisomboonsuk B, Klungthong C,	Levy JW, Velasco JM, Roque VG Jr, Salje H, Macareo LR, Hermann LL, Nisalak A, Srikiatkhachorn	A. High rate of subclinical chikungunya virus infection and association of neutralizing antibody	with protection in a prospective cohort in the Philippines. PLoS Negl Trop Dis.	2015;9(5):e0003764. doi: 10.1371/journal.pntd.0003764.

**2) The headline figure of 40/1,089 (3.7%) of samples had evidence of acute CHIKV infection is probably not the best figure to use due to the way sampling was conducted – virtually all DENV negative samples were tested however only a subset of DENV positive samples were tested. I would instead report the two figures separately - so XX/XX (XX%) of DENV negative samples had evidence of ACI and XX/XX (XX%) of DENV positive samples have evidence of ACI.**

**Response:** Amongst the 40 acute chikungunya infection cases, all of them were from the non-DENV group. None of the 105 confirmed DENV infection had evidence of acute chikungunya infection. To clarify, in the epidemiology sub-section result, we add “all of whom were DENV-negative subjects” throughout the text.

**Line 43-44 now read:** ACI was confirmed in 40/1,089 (3.7%) screened subjects, all of whom were DENV negative.

**Line 204-205 now read:** Evidence of ACI was found in 40/1,089 (3.7%) subjects, all of whom were DENV-negative.

**3) It would be useful to report the month and year of the ACI cases too - maybe even present an epidemic curve.**

**Response:** Thank you for the suggestion. We now present monthly distribution of ACI cases by sites as Figure 3.

**Fig 3**. **Distribution of acute chikungunya infection cases by month and year**

Reviewer #2: the article is well written and presents interesting results.

**Response:** Thank you very much for your review.
